# A multicenter, randomized, active-controlled, clinical trial study to evaluate the efficacy and safety of navigation guided balloon Eustachian tuboplasty

**DOI:** 10.1038/s41598-021-02848-1

**Published:** 2021-12-02

**Authors:** Sung-Won Choi, Se-Joon Oh, Yehree Kim, Min Young Kwak, Myung-Whan Suh, Moo Kyun Park, Chi Kyou Lee, Hong Ju Park, Soo-Keun Kong

**Affiliations:** 1 Department of Otorhinolaryngology-Head and Neck Surgery, Pusan National University School of Medicine, Biomedical Research Institute, Pusan National University Hospital, Gudeok-ro 179, Seo-Gu, Busan, 49241 Republic of Korea; 2 Department of Otorhinolaryngology-Head and Neck Surgery, Asan Medical Center, University of Ulsan College of Medicine, Seoul, Republic of Korea; 3 Department of Otorhinolaryngology-Head and Neck Surgery, College of Medicine, Eulji University, Daejeon, Republic of Korea; 4 Department of Otorhinolaryngology-Head and Neck Surgery, Seoul National University Hospital, Seoul, Republic of Korea; 5 Department of Otorhinolaryngology-Head and Neck Surgery, Seoul National University College of Medicine, Seoul National University Hospital, Seoul, Republic of Korea; 6 Department of Otorhinolaryngology-Head and Neck Surgery, Soonchunhyang University College of Medicine, Cheonan Hospital, Cheonan, Republic of Korea

**Keywords:** Randomized controlled trials, Outcomes research

## Abstract

To assess the safety and efficacy of navigation-guided balloon Eustachian tuboplasty (BET) compared to medical management (MM) alone in patients with chronic Eustachian tube dilatory dysfunction (ETD). This is a prospective, multicenter, 1:1 parallel-group, randomized controlled trial (RCT). It aims to assess the efficacy of navigation-guided BET compared to MM alone in patients with chronic ETD. The primary outcome measure was an improvement in the Eustachian tube dysfunction questionnaire (ETDQ)-7 score at the 6-week follow-up compared with baseline. Secondary outcome measures included changes in the signs and symptoms during the follow-up, changes in the score for each subcategory of ETDQ-7, type of tympanometry, pure tone audiometry, and the availability of a positive modified Valsalva maneuver. Navigation-guided BET was safely performed in all patients. A total of 38 ears of 31 patients (19 ears of 16 patients in the BET group and 19 ears of 15 patients in the control group) completed the planned treatment and 6 weeks of follow-up. More patients in the BET group (1.99 ± 0.85) had less symptomatic dysfunction than in the control group (3.40 ± 1.29) at 6 weeks post-procedure (*P* = 0.001). More patients experienced tympanogram improvement in the BET group at 6 weeks compared to the control group (36.5% vs. 15.8%) with a positive modified Valsalva maneuver (36.6% vs. 15.8%, *P* = 0.014). Additionally, air–bone gap change was significantly decreased in the BET group compared to the control group at the 6-week follow-up visit (*P* = 0.037). This prospective, multicenter, RCT study suggests that navigation-guided BET is a safe and superior treatment option compared to MM alone in patients with chronic ETD.

## Introduction

Eustachian tube dilatory dysfunction (ETD) can lead to severe consequences, including hearing loss, chronic otitis media, and cholesteatoma^1^. The etiology of ETD is attributed to structural as well as functional entities. Endoscopic studies have found that inflammation within the cartilaginous portion of the Eustachian tube (ET) was the most common finding in ETD^2–6^. To date, many studies regarding the treatment of ETD have been conducted^7^. However, the effectiveness of systemic decongestants, antihistamines, nasal topical decongestants, or steroid sprays shows no well-founded benefits for these treatments^8,9^. Since Ockermann et al.^10^ first introduced endoscopic BET in 2010^4^, balloon dilatation of the Eustachian tube (BET, balloon Eustachian tuboplasty) remains a novel treatment method for ETD^11–13^. Nevertheless, the evidence for BET remains elusive. There are few randomized controlled trials (RCTs) regarding this procedure^1,14^. Recently, a relatively new treatment modality has emerged, navigation-guided BET. Navigation-guided BET involves an image-guided navigation balloon catheter that provides surgeons with real-time feedback on the position of the catheter tip during BET. This novel procedure is technically feasible and safe^15^.

The aim of this pilot study was to assess the safety and efficacy of navigation-guided BET compared to medical management (MM) alone in patients with chronic ETD. To examine the efficacy of navigation-guided BET versus medial management alone, the 7-item Eustachian Tube Dysfunction Questionnaire (ETDQ-7) (Table 1) score was used. The ETDQ-7 assesses symptoms as well as pure tone audiometry (PTA), tympanometry, and the ability to perform a Valsalva maneuver^16^. A prospective, multicenter, RCT was conducted with patients who were diagnosed with chronic ETD. The results of a study group and a control group were compared after 6 weeks of treatment to determine improvements.

**Table 1:** The seven-item Eustachian tube dysfunction questionnaire^16^.

Over the past 1 month, how much has each of the following been a problem for you?	No problem		Moderate problem			Severe problem	
1. Pressure in the ears?	1	2	3	4	5	6	7
2. Pain in the ears?	1	2	3	4	5	6	7
3. A feeling that your ears are clogged or “under water”?	1	2	3	4	5	6	7
4. Ear symptoms when you have a cold or sinusitis?	1	2	3	4	5	6	7
5. Crackling or popping sounds in the ears?	1	2	3	4	5	6	7
6. Ringing in the ears?	1	2	3	4	5	6	7
7. A feeling that your hearing is muffled?	1	2	3	4	5	6	7

## Results

### Patient characteristics and demographics

**Figure 1:**
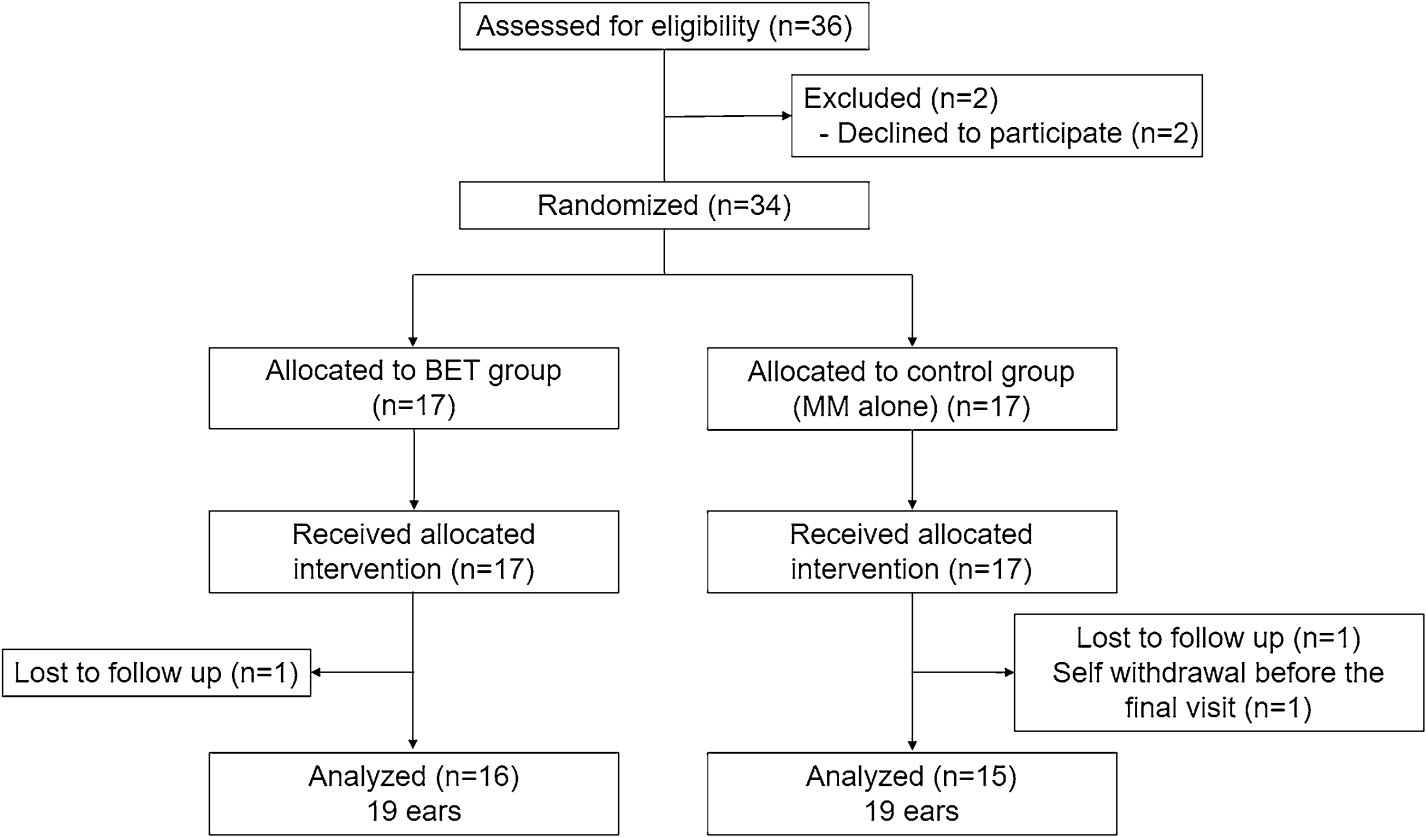
Participants flow diagram.

Among the 36 patients who met the inclusion criteria, 34 patients were enrolled and randomized. Of these, 17 patients were randomly assigned to the BET group, whereas 17 patients were assigned to the control group (MM alone). One patient in the BET group was excluded because the patient was lost to follow-up. Two patients in the control group were excluded because they were lost to follow-up or self-withdrew prior to the final visit. A total of 38 ears of 31 patients (19 ears of 16 patients in the BET group and 19 ears of 15 patients in the control group) completed the planned treatment and 6 weeks of follow-up (Fig. 1). Baseline demographic data of the patients showed no significant differences between the two groups (Table 2).

**Table 2:** Patients baseline demographic characters and results of laboratory tests.

	BET group (n = 16)	Control group (n = 15)	*P* value	Total
**Demographic data**				
Age	44.29 ± 14.88	45.12 ± 16.34	0.878	44.71 ± 15.39
Sex (M:F)	10:6	8:7	0.540	18:13
Height (cm)	167.82 ± 7.92	165.71 ± 9.40	0.482	166.76 ± 8.63
Body weight (kg)	67.38 ± 11.42	64.32 ± 12.74	0.166	65.87 ± 12.13
**Laboratory data**				
SBP (mmHg)	121.53 ± 12.01	125.00 ± 14.00	0.443	
DBP (mmHg)	73.59 ± 9.15	78.47 ± 9.57	0.138	
Pulse rate (/min)	72.71 ± 10.50	75.00 ± 9.22	0.503	
Body temperature (˚C)	36.59 ± 0.22	36.63 ± 0.20	0.569	
WBC (/dl)	6.04 ± 1.04	6.11 ± 1.39	0.861	
RBC (/dl)	4.64 ± 0.43	4.39 ± 0.36	0.081	
Hb (g/dl)	14.47 ± 1.25	13.61 ± 1.56	0.086	
Hct (%)	42.58 ± 3.97	40.71 ± 4.02	0.181	
Plt (×10^3^/ul)	249.06 ± 51.73	252.76 ± 44.72	0.654	
BUN (mg/dl)	12.31 ± 3.84	12.12 ± 3.32	0.875	
Creatinine (mg/dl)	0.78 ± 0.18	0.79 ± 0.12	0.807	
AST (IU/L)	23.18 ± 7.71	22.35 ± 9.60	0.691	
ALT (IU/L)	22.47 ± 12.09	18.76 ± 15.55	0.087	
FBS (mg/dl)	101.59 ± 9.00	106.76 ± 35.02	0.234	
PT (s)	11.25 ± 0.84	11.58 ± 0.60	0.192	
PTT (s)	29.03 ± 3.99	29.80 ± 4.02	0.512	
PT(INR)	0.98 ± 0.08	1.00 ± 0.07	0.308	

ALT, alanine aminotransferase; AST, aspartate aminotransferase; BUN, blood urea nitrogen; DBP, diastolic blood pressure; FBS, fasting blood sugar; Hb, hemoglobin; Hct, hematocrit; INR, international normalized ratio; Plt platelet, PT prothrombin time; PTT, partial thromboplastin time; RBC, red blood cell; SBP, systolic blood pressure; WBC, white blood cell.

### Efficacy of treatment

#### Improvement of ETDQ-7 scores

**Figure 2:**
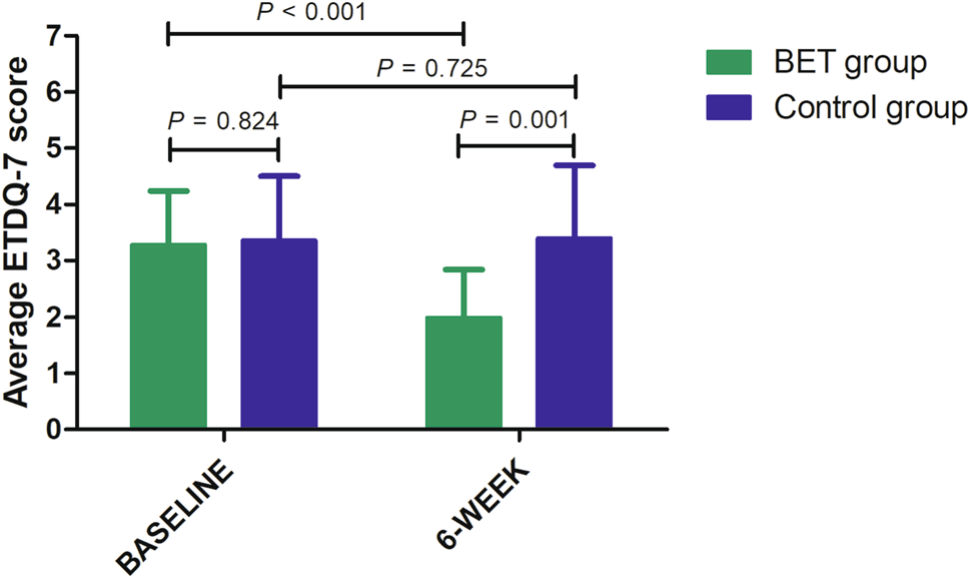
The average ETDQ-7 scores preoperatively and at 6 weeks post-procedure.

There was no significant difference in the average ETDQ-7 scores between the BET group (3.28 ± 0.96) and the control group (3.36 ± 1.14) before surgery (*P* = 0.824). However, more patients in the BET group (1.99 ± 0.85) had less symptomatic dysfunction than those in the control group (3.40 ± 1.29) at 6 weeks post-procedure (*P* = 0.001) (Fig. 2). In addition, in the BET group, the improvement in average ETDQ-7 scores pre- and post-surgery was significantly reduced (*P* < 0.001). However, in the control group, there was no significant difference (*P* = 0.725) (Fig. 2). In the BET group, changes in each of the seven subcategories of the ETDQ-7 score decreased significantly for questions 1, 3, 4, 6, and 7. For questions 2 and 5, the decrease was not significant (Table 3).

**Table 3:** Changes in ETDQ-7 scores and pure tone audiograms in both group.

	Baseline	6 weeks	*P* value
***ETDQ-7 Scores***			
**BET group**			
Question 1	3.06 ± 1.91	1.75 ± 1.18	0.002*
Question 2	2.63 ± 1.54	1.75 ± 1.18	0.079
Question 3	4.44 ± 1.67	2.69 ± 1.82	0.005*
Question 4	3.19 ± 2.17	1.63 ± 1.31	0.012*
Question 5	1.81 ± 1.42	1.13 ± 0.34	0.125
Question 6	2.88 ± 1.89	2.00 ± 1.63	0.039*
Question 7	4.94 ± 1.65	3.00 ± 2.00	0.001*
**Control group**			
Question 1	2.73 ± 1.87	2.53 ± 1.46	0.625
Question 2	1.87 ± 1.19	2.00 ± 1.20	0.698
Question 3	4.53 ± 1.88	4.73 ± 1.91	0.531
Question 4	3.33 ± 2.32	2.53 ± 2.00	0.219
Question 5	2.13 ± 1.96	2.33 ± 2.09	0.625
Question 6	3.60 ± 2.20	4.27 ± 2.12	0.031*
Question 7	5.40 ± 1.50	5.47 ± 1.68	0.633
***Pure tone audiometry (dB HL)***			
**Bone conduction threshold**			
BET group	19.56 ± 12.64	18.94 ± 11.42	0.559
Control group	15.27 ± 13.56	15.67 ± 12.70	0.356
Air conduction threshold			
BET group	31.56 ± 18.68	27.50 ± 15.88	0.139
Control group	23.33 ± 18.66	25.60 ± 18.84	0.678
Air–bone gap change	−4.06 ± 7.93	2.27 ± 6.46	0.037*

*P < 0.05, ETDQ, Eustachian tube dysfunction questionnaire; BET, balloon Eustachian tuboplasty; dB, decibel; HL, hearing level.

#### Changes in the type of tympanometry, pure tone audiometry, and availability of positive a modified Valsalva maneuver

**Figure 3:**
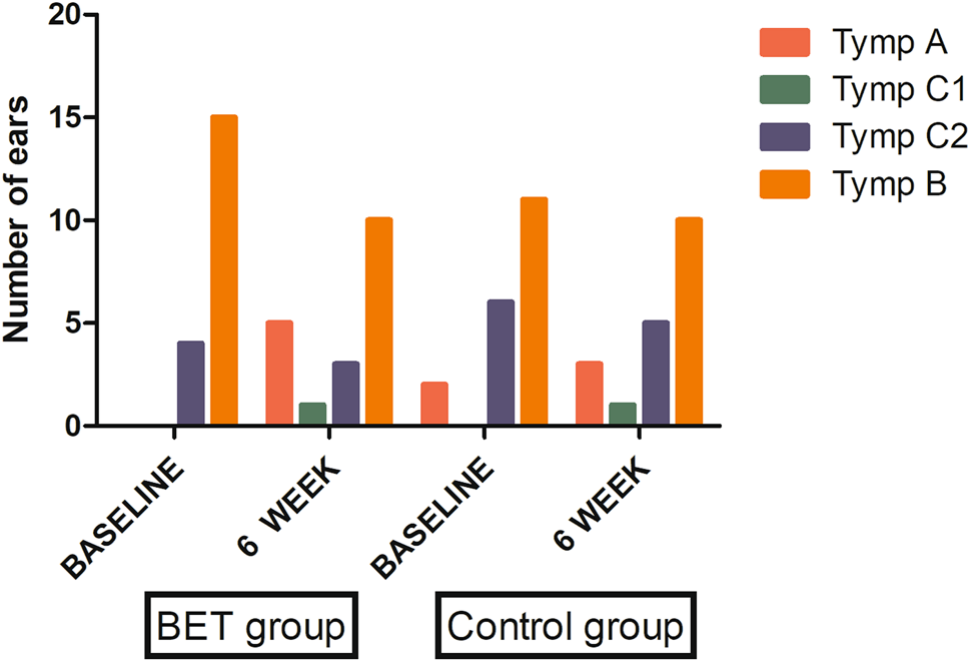
Tympanogram changes of treatment effects preoperatively and at 6 weeks post-procedure.

The ears in the BET group (7/19; 36.8%) showed improvement in the tympanogram (four ears: B to A, one ear: C2 to A, one ear: C2 to C1, and one ear: B to C2) from baseline to the 6-week follow-up compared to 15.8% (3/19) of ears in the control group (one ear: C2 to A, one ear: C2 to C1 and one ear: B to C2) (Fig. 3). Improvement in tympanogram showed B or C2 to C1 or A, or B to C2; note that the two tympanogram C types were: type C1 (the pressure between − 100 and − 199 daPa) and type C2 (the pressure − 200 daPa or less)^17^. Tympanograms remained unchanged in 63.2% of ears in the BET group (12/19) compared to 84.2% in the control group (16/19).

**Figure 4:**
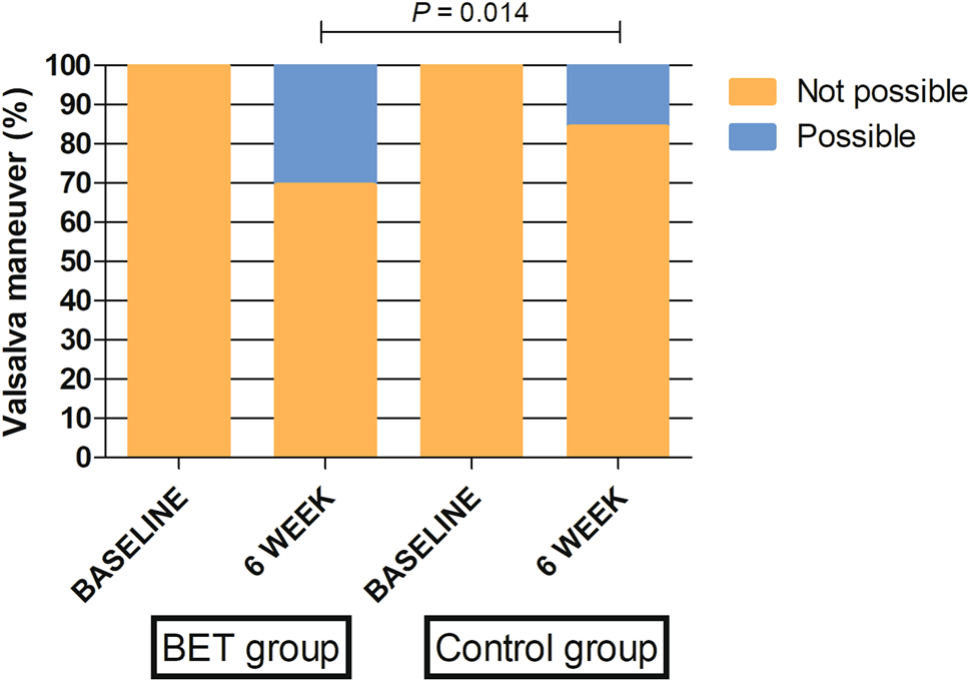
Changes in the positive modified Valsalva maneuver preoperatively and at 6 weeks post-procedure.

Hearing levels were compared between the BET group and the control group and are shown in Table 3. Baseline bone conduction threshold values in the BET and control groups were 19.56 ± 12.64 and 15.27 ± 13.56, respectively (*P* = 0.089). Baseline air conduction threshold values in the BET and control groups were 31.56 ± 18.68 and 27.50 ± 15.88, respectively (*P* = 0.139). There was no significant difference in the hearing level between the BET and control groups prior to surgery. However, the air–bone gap change was significantly decreased in the BET group compared to the control group at the 6-week follow-up visit (*P* = 0.037). In comparison to baseline, there was a 31.6% (6/19) versus 15.8% (3/19) with a positive modified Valsalva maneuver in the BET group compared to the control group at the 6-week follow-up (Fig. 4). The performance of a positive modified Valsalva maneuver at 6-weeks was significantly higher in the BET group (*P* = 0.014).

### Safety data

No procedure-related or device-related serious adverse event was reported through the last follow-up in either the BET group or the control group. There were no postoperative patulous ET or false passage that occurred during BET. In addition, no medication-related adverse events were reported in the control group.

## Discussion

BET has emerged as a new treatment modality for ETD owing to its’ minimally invasive nature^18,19^. The underlying mechanism is hypothesized to be that the submucosal applied pressure causes fibrosis and expansion of the ET^20^. Although BET is widely accepted as a treatment for chronic ETD, the safety and effectiveness of BET compared to MM are not well evaluated. In this study, we compared the safety and effectiveness of BET versus MM alone in patients with chronic ETD. The results of the present study demonstrate that the effectiveness of BET is clinically significant versus MM alone for the treatment of patients with chronic ETD.

In previous studies, very few randomized control studies proved the effectiveness and safety of BET in the treatment of chronic ETD. In the review, only four studies were well-designed randomized control studies that evaluated the effectiveness and safety of BET in the treatment of chronic ETD^1,14,18,19^. Poe et al. showed that more patients experienced tympanogram normalization in the BET group at 6 weeks compared to the control group (MM alone) (51.8% vs. 13.9%, *P* < 0.001) with normalized ETDQ-7 scores (56.2% vs. 8.5%, *P* < 0.001)^1^. Meyer et al., reported a mean change in the overall ETDQ-7 score at 6 weeks. The overall score was −2.9 ± 1.4 for the BET group compared with −0.6 ± 1.0 for the control group (MM alone) (*P* < 0.001)^18^. In addition, two studies showed that the long-term outcomes of BET demonstrate significant durability for 52 weeks or longer than 2 years^14,19^. In this study, the effectiveness and safety of BET were similar to those of previous studies. The primary endpoint was met. In the BET group (1.99 ± 0.85), symptomatic dysfunction was less than that in the control group (3.40 ± 1.29) at 6 weeks post-procedure (*P* = 0.001). In addition, in the BET group, the improvement in average ETDQ-7 scores pre- and post-surgery was significantly reduced (*P* < 0.001). Although, in the control group there was no significant difference (*P* = 0.725). In this study, the secondary outcome measures included changes in the type of tympanometry, pure tone audiometry, and the availability of a positive modified Valsalva maneuver. Previous studies have also reported changes in the type of tympanometry and availability of a positive modified Valsalva maneuver. However, no single study has reported pre- and post-surgery results of PTA. In the present study, more patients experienced tympanogram improvement in the BET group at 6 weeks compared to the control group (36.5% vs. 15.8%) with a positive modified Valsalva maneuver (36.6% vs. 15.8%, *P* = 0.014). In this study, we compared the PTA results pre- and post-BET, unlike other previous studies. We included hearing tests and reported that the air–bone gap change was significantly decreased in the BET group compared to the control group at the 6-week follow-up visit (*P* = 0.037). According to the results of this study, BET demonstrates an improvement not only in subjective symptoms such as the ETDQ-7, but also an improvement in objective hearing gain.

Although our study demonstrated the advantages of using navigation-guided BET, it has some limitations. First, tympanograms remained unchanged in 63.2% of ears in the BET group (12/19) compared to 84.2% in the control group (16/19). There was no significant difference in the tympanogram normalization between the two groups. Annand et al. reported that the onset of benefits from BET could be delayed by 52 weeks^14^. Therefore, it is thought that long-term follow-up is necessary rather than a 6-week follow-up. There may be a delayed onset benefit in this study. In the absence of long-term follow-up for comparison, tympanogram normalization should be examined in future studies. Second, previous studies have reported that the increase in the number of ears with a Valsalva maneuver post-BET was 70–80%^21–23^. However, in this study, the increase was 31.6% (6/19) versus 15.8% (3/19) with a positive modified Valsalva maneuver in the BET group compared to the control group at the 6-week follow-up, respectively. The performance of a positive modified Valsalva maneuver at 6-weeks was significantly high in the BET group. However, this was at a lower rate compared to previous studies. These results might be affected by the small number of patients included in this study and the inability of patients to perform a Valsalva maneuver. This is possible that there were more severe cases compared to other previous studies.^1,14^ To evaluate severity of ETD symptoms, we used the validated ETDQ-7 scores survey in this study. The preoperative average ETDQ-7 is similar to or lower than that of other previous studies^1,14^, so it is unlikely that there were more severe cases compared to other previous studies. Third, patients with adhesive otitis media despite MM may occur not only in cases of chronic ETD but also in cases of sniff-type patulous Eustachian tube. Future research should clearly classify these points in the inclusion criteria. Also, the inclusion criteria of previous studies were not the same^7^. For these reasons, further studies with larger sample sizes are required to validate our results.

However, the present lack of diagnostic consensus does not deny the beneficial effects of BET in patients with chronic ETD. In view of the results of this prospective, multicenter, RCT study, BET is applicable in patients with medically refractory chronic ETD aged 19 years or older regardless of the severity of symptoms.

In summary, there were no procedure-related complications. Navigation-guided BET was technically successful and safely performed in all patients. During all the BET procedures, we were able to confirm the insertion depth of the catheter and establish awareness of the proximity of the internal carotid artery^15^. This prospective, multicenter, RCT study suggests that navigation-guided BET is a superior treatment option compared to MM alone in patients with chronic ETD. The beneficial effects on subjective symptoms and objective findings include: performing a positive modified Valsalva maneuver and decreased air–bone gap change. This demonstrates a significant clinical relevance through 6 weeks.

## Materials and methods

### Trial design, setting and interventions

This was a prospective, multicenter, 1:1 parallel-group RCT to assess the efficacy of navigation-guided BET compared to MM alone in patients with chronic ETD. This study was conducted in four tertiary hospitals in South Korea. The initial enrolment target number was 36. The present study was a pilot study. The sample size was determined based on the eligible number of patients that were available from four tertiary hospitals between April 2019 and December 2020 (Fig. 1). Patients with chronic ETD were randomly assigned to either the BET group (n = 17) or the control (MM alone) group (n = 17). Randomization was performed using a concealed, computer-generated list of management assignments. This was based on the predetermined simple randomization schedule provided by the central data management committee. The randomized patients were followed for all clinical endpoints and serious adverse events. The primary and several secondary endpoint events confirmed by central adjudication were blinded to the study assignments. Prior to treatment, the ETDQ-7 score was used to assess symptoms, pure tone audiometry (PTA), tympanometry, and the ability to perform a Valsalva maneuver. Demographic data were obtained and clinical laboratory tests such as hematology and serum chemistry were performed.

In the BET group, BET analysis was performed under general anesthesia. Each patient underwent BET using both the Naviloon (balloon and guide catheter) (Mega Medical, Seoul, Korea) and the Mega Navigation (image-guided navigation system; Mega Medical, Seoul, Korea). The balloon was inflated once with sterile water to a target pressure of up to 12 atm for 2 min, deflated and then reinflated for 1 min^15^. Methods related to detailed description of this navigation guided balloon Eustachian tuboplasty are described in supplementary methods and supplementary Fig. 1. In the control (MM alone) group, subjects in the investigational cohort began a fluticasone furoate (Avamys®) nasal spray regimen consisting of two sprays to each nostril once per day (110 µg/day)^24,25^. In addition, bepotastine besilate (Talion®) tablets (20 mg/day, twice daily) and ranitidine hydrochloride (Curan®) tablets (300 mg/day, twice daily) were administered orally from day 1 to 6 weeks.

Patients completed follow-up visits 6 weeks after the initiation of treatment. After completion of the clinical trial, the results were revealed and analyzed by the outcome evaluators.

### Outcome parameters

The primary outcome measure was an improvement in the ETDQ-7 score at the 6-week follow-up compared with the baseline score^16^. ETDQ-7 scores at 6 weeks after treatment were compared with those at baseline. Subjects were said to have ‘improved’ if the total scores had decreased and ‘aggravated’ if the total scores increased. A blinded, independent evaluator, irrelevant to the patient’s treatment, reviewed all the ETDQ-7 score survey responses.

Secondary outcome measures included changes in signs and symptoms during the follow-up, changes in the score for each subcategory of ETDQ-7, type of tympanometry, PTA as the average of four frequencies (0.5, 1, 2, and 3 kHz), and availability of a positive modified Valsalva maneuver (nose blow + swallow).

Safety was assessed by physical examination and interviews during the study period. The normality of laboratory findings was determined. Any clinically significant abnormality was evaluated and recorded on an adverse event report form.

### Ethical approvals, registrations, and patient consents

This clinical trial was conducted according to the study protocol, which was approved by the Institutional Review Board (IRB) of Pusan National University Hospital (D-1903-020-077), Asan Medical Center (2018-2213), Seoul National University Hospital (D-1812-087-995), and Soonchunhyang University Cheonan Hospital (2018-12-020), and the study was conducted adhering to the Helsinki Declaration of 1975 as revised in 2013. This study was performed in accordance with Good Clinical Practice Guidelines and stuck to the applicable Consolidated Standards of Reporting Trials (CONSORT) guidelines. Written informed consent was obtained from all patients before enrolment. The study protocol was registered at the Clinical Research Information Service (KCT0005627; data of registration 24/11/2020).

### Patients

The inclusion criteria for this study were designed to include patients with confirmed chronic ETD at the participating tertiary hospitals. Patients were ≥ 19 years of age. A diagnosis of chronic ETD was based on at least one of the following protocols: (1) patients reported symptoms (ear pain, ear pressure, tinnitus, cracking or popping in ears, muffled hearing, and feeling that their ears were clogged) and at least three or more symptoms lasting more than 12 months, (2) patients with repeated serous otitis media despite MM, or (3) patients with adhesive otitis media despite MM. In addition, absence of internal carotid artery (ICA) dehiscence into the ET lumen was confirmed by a computed tomography (CT) scan.

The exclusion criteria for this study were as follows: (1) ICA dehiscence confirmed by CT; (2) diagnosis of patulous ET; (3) tympanic membrane perforation or presence of a tympanostomy tube; (4) diagnosis of Meniere’s disease or chronic rhinosinusitis; (5) anatomically difficult to access ET through the nasal cavity; (6) history of major head or neck surgery within 4 months; (7) active chronic or acute otitis media; (8) pregnancy, breast-feeding, or child-bearing potential; (9) severe hepatic dysfunction (AST, ALT is 2.5 3 times higher than the normal upper limit); (10) severe renal dysfunction (Cr > 2.0 mg/dl); (11) history of malignant tumors including leukemia and lymphoma within the past 5 years; (12) patients who had participated in another clinical trial of a drug or medical device within 4 weeks prior to screening.

### Statistical analysis

To calculate the number of valid participants in this study, G*Power software version 3.1.9.2 for Windows was used to calculate the sample size. Power (1- ß error) was set at 0.8 with an error of 0.05 (Level of significance, α = 0.05 Z_α/2_ = 1.96, Power of the test, 1-β = 0.8, Z_1- β_ = 0.8416, *D*_*t*_ − *D*_*c*_ = 1.3, σ = 1.3). The result of power calculation showed that a sample size of 19 patients in each group would give us the ability to confirm the differences. According to the references^1,14^, the average difference in the ETDQ-7 score of the experimental group was 2.15, and the average difference in the ETDQ-7 score of the control group was 0.85 that indicate a significant decrease. Therefore, the difference of the amount of score change between the experimental and the control group was investigated as 1.3.

The significance of the difference between groups was tested using an independent two-sample *t*-test or Mann–Whitney *U* test for continuous data and Pearson's chi-square test or Fisher's exact test for categorical data. For the significance of changes within each group, the paired t-test or Wilcoxon signed rank test for continuous variables and McNemar's chi-square test for categorical data were used. In the case of missing values, the missing values were processed using the mean substitution method for continuous data among the validity data. Statistical analysis was performed using SAS version 9.4 (SAS Institute, Cary, NC, USA) and *P* < 0.05 was considered significant.

## Supplementary Information

Supplementary Information.

## Data Availability

The datasets generated during and/or analyzed during the current study are available from the corresponding author on reasonable request.
