# Supplementary material for: A multicenter, randomized, active-controlled, clinical trial study to evaluate the efficacy and safety of navigation guided balloon Eustachian tuboplasty
Source: Sci Rep. 2021 Dec 2;11:23296. doi: 10.1038/s41598-021-02848-1 (PMC8639820; doi:10.1038/s41598-021-02848-1)
Supplement: Supplementary file 1 — Supplementary Information. [file 41598_2021_2848_MOESM1_ESM.docx]

**Supplementary Information**

**A multicenter, randomized, active-controlled, clinical trial study to evaluate the efficacy and safety of navigation guided balloon Eustachian tuboplasty**

Sung-Won Choi^1^, Se-Joon Oh^1^, Yehree Kim^2^, Min Young Kwak^3^, Myung-Whan Suh^4^, Moo Kyun Park^5^, Chi Kyou Lee^6^, Hong Ju Park^2^, Soo-Keun Kong^1 *^

**Supplementary Methods**

**Balloon dilatation using the image-guided navigation balloon catheter**

The balloon catheter is designed to be inserted and inflated in the cartilaginous portion of the ET for treatment of ETD. The balloon catheter consists of a flexible proximal shaft, an actuator for advancing and retracting the balloon catheter, a rigid shaft that passes through the guide catheter, a balloon (length 16 mm, diameter 6 mm at 12 atm) at the distal end of the shaft for ET dilation, and a distal atraumatic tip that contains an electromagnetic navigation sensor. The NET-Navigation target localization error for surface registration is less than 2mm. Although the balloon catheter was equipped with a navigation system, the catheter also has a black proximal endoscopic marker to aid in positioning under direct endoscopic visualization (Supplementary Figure 1). The guide catheter is designed to provide a means to access the ET. Unlike other guide catheters, this device consists of a flexible shaft with a central lumen instead of a rigid shaft. If anatomical variations such as deviated nasal septum, inferior turbinate hypertrophy, or narrow nasal cavity were present in the patient, surgeons can easily bend the flexible shaft according to the anatomical variation during BET. The catheter tip was used as a registration probe and contacted the face during the skin tracing process. All procedures were performed with endoscopic assistance (30 degree view angle). A balloon was slowly passed through the ET lumen until it reached the isthmus with the cartilaginous portion of the ET, which was confirmed simultaneously by the navigation system. The balloon was inflated once with sterile water to a target pressure of up to 12 atm for 2 minutes, then deflated for 1 minute, and then reinflated for 1 more minute^15^.

**Supplementary Figure**


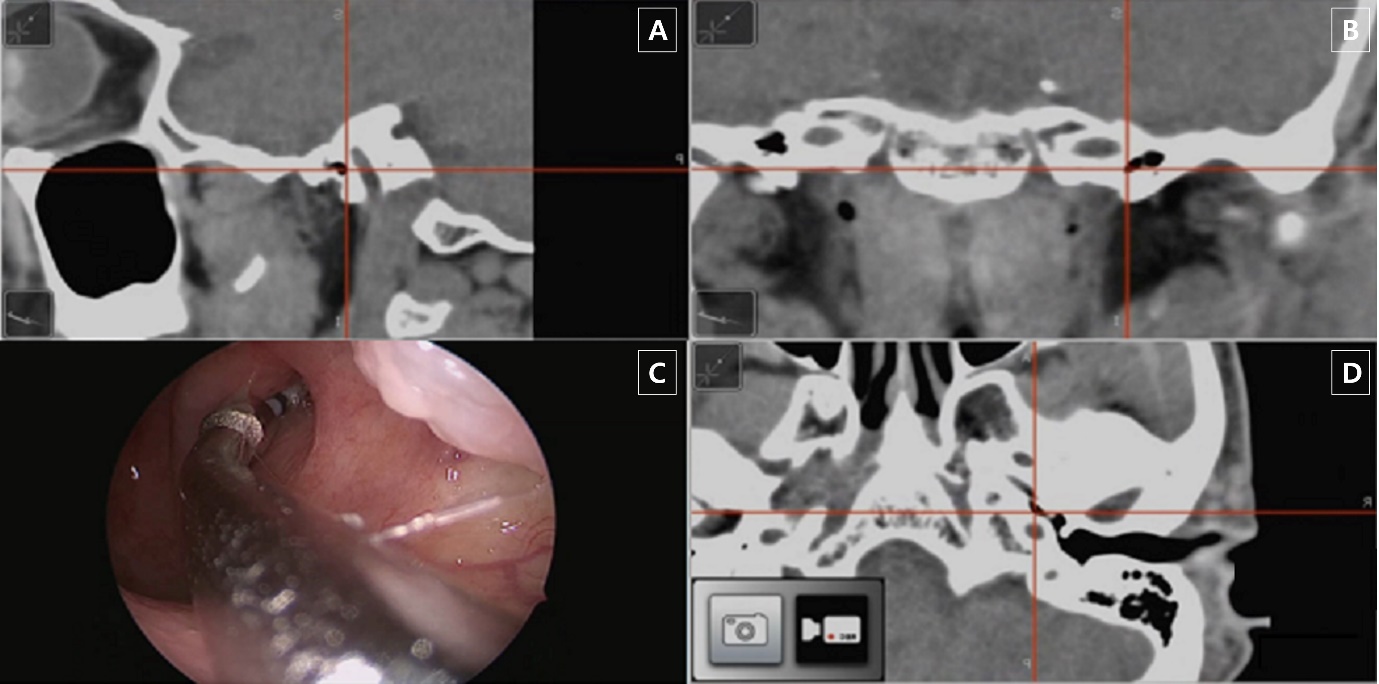


**Supplementary Fig. 1.** Intraoperative view showing the insertion depth of the catheter tip as confirmed during a balloon Eustachian tuboplasty. (A) Sagittal view, (B) Coronal view, (C) Endoscopic image of catheter tip insertion over a black proximal endoscopic marker. (D) Axial view.
